# Urinary N‐Telopeptide as Predictor of Onset of Menopause‐Related Bone Loss in Pre‐ and Perimenopausal Women

**DOI:** 10.1002/jbm4.10116

**Published:** 2018-12-30

**Authors:** Albert Shieh, Gail A Greendale, Jane A Cauley, Carrie Karvonen‐Gutierrez, Joan Lo, Arun S Karlamangla

**Affiliations:** ^1^ Division of Geriatrics Department of Medicine David Geffen School of Medicine at University of California Los Angeles, Los Angeles CA USA; ^2^ Department of Epidemiology Graduate School of Public Health University of Pittsburgh Pittsburgh PA USA; ^3^ School of Public Health University of Michigan Ann Arbor MI USA; ^4^ Kaiser Permanente Division of Research Oakland CA USA

**Keywords:** MENOPAUSE, BIOCHEMICAL MARKERS OF BONE TURNOVER, OSTEOPOROSIS, DXA, GENERAL POPULATION STUDIES

## Abstract

The menopause transition (MT) is a period of rapid bone loss and has been proposed to be a time‐limited window for early intervention to prevent permanent microarchitectural damage and reduce the risk of subsequent fracture. To intervene early, however, we first need to be able to determine whether menopause‐related bone loss is about to begin, in advance of substantial bone loss. The objective of this study was, therefore, to assess whether urinary N‐telopeptide (U‐NTX) in pre‐ or early perimenopause can predict the onset of menopause‐related bone loss. Repeated U‐NTX measurements were obtained during pre‐ and early perimenopause in 1243 participants from the Study of Women's Health Across the Nation (SWAN). We examined the ability of U‐NTX to predict the onset of significant menopause‐related bone loss (categorical outcome, yes versus no) at the lumbar spine (LS) and femoral neck (FN), defined as annualized bone mineral density (BMD) decline at a rate faster than the smallest detectable change in BMD over the 3 to 4 years from the time of U‐NTX measurement. Adjusting for age, race/ethnicity, body mass index, urine collection time, starting BMD, and study site in multivariable, modified Poisson regression, every standard deviation increment in U‐NTX, measured at baseline in early perimenopausal women, was associated with an 18% and 22% greater risk of significant bone loss at the LS (*p* = 0.003) and FN (*p* = 0.003), respectively. The area under the receiver‐operator curve for predicting LS and FN bone loss was 0.72 and 0.72, respectively. In mixed‐effects analysis of all repeated measures of early perimenopausal U‐NTX over follow‐up, U‐NTX predicted onset of bone loss at the LS (*p* = 0.002) but not at the FN. We conclude that U‐NTX can be used early in the MT to determine if a woman is about to experience significant LS bone loss before there has been substantial skeletal deterioration. © 2018 The Authors. *JBMR Plus* is published by Wiley Periodicals, Inc. on behalf of the American Society for Bone and Mineral Research.

## Introduction

Osteoporosis is characterized by skeletal fragility resulting from decreased bone mineral density (BMD) and impaired bone quality.^1^ In women, menopause transition (MT)‐related BMD decline accelerates 1 year before the final menstrual period (FMP) and slows slightly in postmenopause.^2^ This loss in BMD during the menopause transition (MT) is accompanied by damage to trabecular microarchictecture that may increase fracture risk.^3^, ^4^ Within the first decade after menopause, vertebral fracture incidence increases.^5^, ^6^ This suggests that the MT is a critical period to intervene to prevent rapid bone loss and permanent microarchitectural deterioration.^7^ To do so, however, we first need to be able to recognize when women are about to lose a significant amount of bone so that they can be targeted for intervention before substantial loss. Although we know that MT‐related BMD decline accelerates around 1 year before the FMP,^2^ an FMP‐based approach to recognizing the onset of significant bone loss is not feasible in real time. This is because the onset time point can only be determined retrospectively 1 year after the FMP has passed, when the FMP can be recognized and dated.

MT‐related BMD decline is driven by an increase in bone resorption.^8^ Prior studies have reported that the bone resorption marker, urinary N‐terminal telopeptide of type I collagen (U‐NTX), begins to increase less than 1 year before the onset of MT‐related bone loss.^2^, ^8^ We, therefore, designed this study to answer the following two questions: 1) Can U‐NTX measured when a woman is in her mid‐40s to early 50s and either premenopausal (ie, still having regular menstrual cycles) or in early perimenopause (ie, starting to have irregular menstrual cycles but with no gaps of 3 months or longer) be used to determine if she is about to begin to experience significant MT‐related bone loss; and 2) Is the ability of U‐NTX to predict the onset of significant MT‐related bone loss different when measured in premenopause versus early perimenopause.

Answering the first question will tell us if age alone should trigger measurement of bone resorption markers for determining the onset of MT‐related bone loss, and the second will tell us if waiting for early perimenopause to measure bone resorption markers will improve the determination and be more efficient. We used data from the Study of Women's Health Across the Nation (SWAN), a longitudinal study of the MT in a multi‐ethnic, community‐based cohort of participants with annual measurements of U‐NTX and BMD, to answer both questions.

## Materials and Methods

SWAN is a multi‐center, community‐based, longitudinal cohort study of the MT. At baseline, participants were aged 42 to 52 years, premenopausal (menstruating 3 months before screening without change in menstrual regularity in the past year) or early perimenopausal (menstruating 3 months before screening with decreased regularity in the past year), had an intact uterus with one or two ovaries, were not pregnant or lactating, and were not taking sex steroid hormones. The entire SWAN cohort included 3302 participants from seven sites: Boston, MA; Chicago, IL; Detroit, MI; Pittsburgh, PA; Los Angeles, CA; Newark, NJ; and Oakland, CA. The SWAN Bone Cohort included 2413 participants from five sites (the Chicago and Newark sites did not perform bone assessments). Among SWAN Bone Cohort participants, the bone resorption marker, U‐NTX, was measured at baseline and annually thereafter until the eighth annual follow‐up visit. BMD was measured at baseline and annually thereafter. Participants gave written informed consent, and sites obtained institutional review board approval.

### Study sample

Of the 2413 bone cohort participants, 70 women were excluded because they did not have U‐NTX measurement at their baseline visit. An additional 1100 women were excluded because we could not determine whether they had lost bone over the next 3 to 4 years, either because they were missing baseline or follow‐up BMD data or because they initiated bone‐modifying medications between the two DXA scans. This left us with a base study sample of 1243 women.

### Predictors

U‐NTX was measured from a non‐first voided urine obtained before 10 a.m. Specimens were stored at −20°C to −80°C at local sites for up to 1 month until shipment to the Central Lab (Medical Research Laboratories, Highland Heights, KY, USA). At the Central Lab, all samples were stored at −80°C. U‐NTX was measured using the Osteomark competitive inhibition enzyme immunoassay (nM BCE; Osteomark, Ostex International Inc., Seattle, WA, SUA; interassay coefficient of variation [CV] <12%; intra‐assay CV <8%). Urinary creatinine was measured using the Cobas Mira autoanalyzer (mM; Horiba ABX, Montpellier, France; interassay CV 4.1%; intra‐assay CV 0.6%). U‐NTX was normalized by urinary creatinine and expressed in nM BCE/mM Cr.^(8,9)^

### Outcomes

Lumbar spine (LS) and femoral neck (FN) BMD were measured by dual‐energy X‐ray absorptiometry (DXA; Hologic [Waltham, MA, USA] QDR 2000 at Pittsburgh and Oakland sites; Hologic QDR 4500A at Boston, Los Angeles, and Michigan sites). Cross‐site calibration was performed by circulating an anthropomorphic spine phantom. Standard quality‐control phantom scans were performed before each BMD measurement session. These were used to adjust for machine drift when necessary. The Pittsburgh and Oakland sites upgraded from the 2000 to 4500A models at follow‐up visit 8. These sites scanned 40 women on both their old and new machines to develop cross‐calibration regression equations.^2^, ^9^

Annualized BMD decline rate was calculated as the percentage of BMD decline from time of U‐NTX measurement to the first BMD measurement 3 to 4 years later, divided by the number of intervening years. We calculated BMD decline over a 3‐ to 4‐year period to allow the change in BMD to be sufficiently large to exceed the precision error in DXA measurements.^10^, ^11^ We determined that a woman was losing significant bone (categorical outcome, yes/no) if the annualized BMD decline rate was faster than a prespecified threshold. We used two different BMD decline rate thresholds to infer significant bone loss. The first was the site‐specific least significant change (LSC) in BMD divided by the median number of intervening years between BMD measurements in the study sample. The LSC for a measure depends on the precision error in its measurement and is the smallest amount of change that would be statistically significantly different from no change at 2‐sided type I error (alpha) of 5%.^10^, ^12^, ^13^ The LSC is recommended for defining whether a true physiologic change in BMD has occurred in clinical practice and is calculated as 2.77 times the CV for the measurement.^10^ In SWAN, each of the five Bone Cohort sites performed duplicate BMD measurements at the LS and FN in five women, with complete repositioning (25 duplicate measurements in total). From these measurements, the short‐term in vivo precision error was calculated using the root mean square SD approach recommended by the International Society for Clinical Densitometry. Because the CV for LS and FN BMD measurements in SWAN were 1.4% and 2.2%, respectively,^2^ and the median number of years between BMD measurement was 3.2 in the study sample, the LSC‐based BMD decline rate threshold was 1.23% per year for LS and 1.93% per year for FN. The second threshold for detecting significant bone loss was based on the distribution of BMD change rates in SWAN participants who were 5 or more years before the FMP and can be presumed to have relatively stable BMD (stable group).^2^ After calculating the ongoing rates of change in BMD in these women, we categorized a rate of change in BMD that was lower (more negative) than the 5th percentile of the site‐specific distribution as significant bone loss (–1.59% per year for LS, −1.86% per year for FN).

### Covariates

Body mass index (BMI) was calculated from weight and height measurements (BMI = weight in kilograms / [height in meters]^2^). Menopause transition status was determined by menstrual bleeding patterns. Premenopause was defined as no change in menstrual regularity in the past year. Early perimenopause was defined as decreased regularity in menstrual bleeding but with no gap of 3 months or more.

### Statistical analysis

The first set of analyses were designed to answer whether U‐NTX in pre‐ or early perimenopausal women, 42 to 52 years old, can help infer if a woman will begin to experience significant bone loss in the next 3 to 4 years. We used multivariable modified Poisson regression with U‐NTX at SWAN baseline as primary predictor and significant bone loss (yes/no) from time of U‐NTX measurement to 3 to 4 years later as outcome and adjusted for MT stage (pre‐ versus early perimenopause), age, race/ethnicity (white, African American, Chinese, Japanese), BMI, urine collection time (to account for diurnal variation in bone turnover markers), starting BMD, and study site as covariates and robust estimation of standard error.^14^ We used modified Poisson regression instead of logistic regression because bone loss is not a rare outcome in this group and because the former approach is more robust to model misspecification.^13^ Separate analyses were conducted for LS bone loss and FN bone loss, each defined using two BMD decline rate thresholds (as described above).

To answer whether the ability of U‐NTX to predict the onset of significant MT‐related bone loss is different when measured in premenopause versus early perimenopause, we first repeated the above analyses after stratifying the study sample by MT stage at SWAN baseline (720 premenopausal, 523 early perimenopausal). Because our ability to detect the hypothesized associations could be limited by the reduced sample size in each stratum, we also conducted repeated measures, mixed‐effects analyses using all premenopausal and early perimenopausal U‐NTX measurements (from baseline and all follow‐up SWAN visits). We used mixed‐effects, modified Poisson regression to separately model premenopausal U‐NTX and early perimenopausal U‐NTX as predictors of significant bone loss over the next 3 to 4 years, while accounting for within‐woman correlations between repeated measures and adjusting for the same covariates as in the baseline analysis (age, race/ethnicity, BMI, urine collection time, starting BMD, and study site). Time‐varying covariates (age, BMI, starting BMD, and urine collection time) were assessed at the time of U‐NTX measurement. As in the baseline analyses, only the current level of U‐NTX was used to predict bone loss; prior measurements were not incorporated in the prediction models. The repeated measures analysis of premenopausal U‐NTX used 1386 observations from 720 women who were in premenopause at SWAN baseline. For the repeated measures analysis of early perimenopausal U‐NTX, we had 2877 observations from 1033 women. This included 523 women who were in early perimenopause at SWAN baseline, plus 510 women who were premenopausal at baseline and had at least one SWAN follow‐up visit in early perimenopause.

For both sets of analyses described above, we conducted parallel sensitivity analyses using the same statistical approach, primary predictors, and covariates, but with significant bone loss (yes/no) defined as a BMD decline rate that was faster than the site‐specific LSC expressed as absolute change in BMD (instead of percent change in BMD). We performed these sensitivity analyses because a given absolute change in BMD will correspond to different percent changes depending on starting BMD. In SWAN, the CVs for LS and FN BMD measurements in absolute terms were 0.012 and 0.014 g/cm^2^, respectively.^2^ With the median number of years between BMD measurements in the study sample being 3.2, the absolute LSC‐based BMD decline rate thresholds were thus 0.016 (LS) and 0.017 (FN) g/cm^2^ per year.

## Results

### Participant characteristics at SWAN baseline

In the baseline U‐NTX analytic sample, mean age was 46.0 years (range 42.0 to 52.8 years). Nearly half were white, 17.8% African American, 14.7% Chinese, and 6.4% Japanese. The majority (57.9%) were premenopausal (Table 1). Mean decline in BMD was faster in the LS than in the FN, and more women experienced significant bone loss in the LS over the next 3 to 4 years from SWAN baseline than in the FN, regardless of which BMD decline rate threshold was used to categorize the onset of bone loss (LSC‐based or distribution‐based).

**Table 1:** Descriptive Statistics^a^ for Analytic Sample at Study Baseline^b^ (Study of Women's Health Across the Nation [SWAN])

	Pre‐ and early perimenopausal subjects *N* = 1243	Premenopausal subjects *n* = 720	Early perimenopausal subjects *n* = 523
Age (years)	46.3 (2.7)	46.0 (2.6)	46.8 (2.7)
Race/ethnicity			
African American	250 (20.1)	128 (17.8)	122 (23.3)
White	621 (50.0)	362 (50.3)	259 (49.5)
Chinese	175 (14.1)	106 (14.7)	69 (13.2)
Japanese	197 (15.8)	124 (17.2)	73 (14.0)
Body mass index (kg/m^2^)	26.7 (6.5)	26.4 (6.4)	27.1 (6.6)
N‐telopeptide, urine (nM BCE/mM Cr)	33.5 (14.6)	33.0 (13.5)	34.1 (16.0)
Annualized percent change in bone mineral density			
Lumbar spine (% per year)	−0.3 (1.2)	−0.2 (1.1)	−0.6 (1.3)
Femoral neck (% per year)	−0.2 (1.2)	−0.1 (1.2)	−0.4 (1.3)
Losing bone from baseline to 3 to 4 years later (least significant change‐based threshold)^c^			
Lumbar spine	254 (20.6)	107 (15.1)	147 (28.3)
Femoral neck	92 (7.4)	36 (5.0)	56 (10.8)
Losing from baseline to 3 to 4 years later (distribution‐based threshold)^d^			
Lumbar spine	189 (15.4)	78 (11.0)	111 (21.4)
Femoral neck	103 (8.3)	39 (5.5)	64 (12.3)

^a^ Count (percentage) for categorical variables; mean (standard deviation) for continuous variables. All variables (other than rate of change) were measured at SWAN baseline visit.

^b^ *N* = 1243. All participants were pre‐ or early perimenopausal at SWAN baseline.

^c^ Significant bone loss (yes/no) using a least significant change‐based threshold was defined as an annualized lumbar spine (LS) or femoral neck (FN) bone mineral density (BMD) decline rate that was faster than 1.23% per year in the LS and 1.93% per year in the FN.

^d^ Significant bone loss (yes/no) using a distribution‐based threshold was defined as an annualized LS or FN BMD decline rate that was faster than 1.59% per year in the LS and 1.86% per year in the FN.

**Figure 1:**
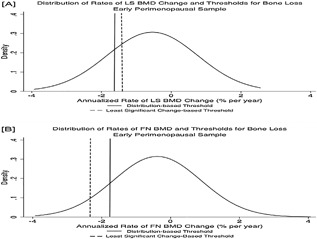
Distribution of rates of change in bone mineral density and thresholds for bone loss at the lumbar spine (*A*) and femoral neck (*B*) among premenopausal women. BMD decline rates to the left of the lines indicating the least significant change‐ and distribution‐based thresholds were considered to reflect bone loss.

**Figure 2:**
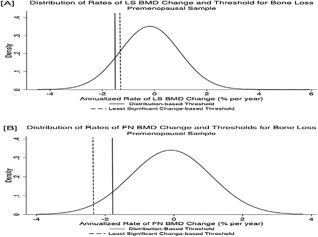
Distribution of rates of change in bone mineral density and thresholds for bone loss at the lumbar spine (*A*) and femoral neck (*B*) among early perimenopausal women. BMD decline rates to the left of the lines indicating the least significant change‐ and distribution‐based thresholds were considered to reflect bone loss. A greater proportion of early perimenopausal women were considered to be losing bone at the lumbar spine (LS) and femoral neck (FN) compared with premenopausal women.

U‐NTX at baseline was normally distributed (median U‐NTX was 31.5 BCE/mM Cr, and the interquartile range [IQR] was from 23.2 to 40.8 BCE/mM Cr). Mean U‐NTX at baseline was similar in early peri‐ and premenopausal women (34.1 versus 33.0 BCE/mM Cr, *p* = 0.2). However, the SD of U‐NTX was larger in early perimenopause (Table 1). Consistent with this, BMD declined faster in early perimenopausal than in premenopausal women over the next 3 to 4 years at both the LS (mean change −0.6% versus −0.2% per year, *p* < 0.001) and FN (mean change −0.4 versus −0.1% per year, *p* < 0.001). A greater proportion of early perimenopausal than premenopausal women was therefore categorized as losing significant bone using either threshold: 28.3 versus 15.1% (*p* < 0.001) in the LS, 10.8 versus 5.0% (*p* < 0.001) in the FN using LSC‐based thresholds, and 21.4 versus 11.0% in the LS (*p* < 0.001), 12.3 versus 5.5% in the FN (*p* < 0.001) using distribution‐based thresholds (Figs.1 and 2).

### U‐NTX as predictor of onset of MT‐related bone loss; SWAN baseline analyses

Greater U‐NTX at SWAN baseline (when participants were aged 42 to 52 years and pre‐ or early perimenopause) was independently associated with greater risk of bone loss onset over the next 3 to 4 years, after adjusting for MT stage, age, BMI, race/ethnicity, urine collection time, starting BMD, and study site in modified Poisson regression (Table 2). Each standard deviation (SD) increment in U‐NTX was associated with a 12% increment in risk of significant LS bone loss (*p* = 0.03) defined as LS BMD decline faster than the LSC‐based threshold (1.23% per year), or 19% increment (*p* = 0.04) using the distribution‐based threshold (1.59% per year). In contrast, baseline U‐NTX was not significantly associated with risk of significant FN bone loss over the next 3 to 4 years for loss defined with either the LSC‐ or distribution‐based thresholds (Table 2).

**Table 2:** Associations Between Urinary N‐Telopeptide Measured at SWAN Baseline and Significant Bone Loss Over the Next 3 to 4 Years

	Bone loss by least significant change‐based threshold^a^		Bone loss by distribution‐based threshold^b^	
Full baseline sample (pre‐ and early perimenopausal women, *N* = 1243)				
	Mean (95% CI)^c^	*p* Value	Mean (95% CI)^c^	p Value
Lumbar spine	1.12 (1.02, 1.23)	0.01	1.19 (1.01, 1.39)	0.04
Femoral neck	1.20 (1.04, 1.37)	0.1	1.14 (0.99, 1.31)	0.05
Stratified baseline analysis				
Premenopausal women (*n* = 720)				
	Mean (95% CI)^d^	*p* Value	Mean (95% CI)^d^	*p* Value
Lumbar spine	1.06 (0.89, 1.26)	0.4	1.05 (0.86, 1.28)	0.6
Femoral neck	1.13 (0.79, 1.63)	0.4	1.15 (0.80, 1.64)	0.3
Early perimenopausal women (*n* = 523)				
	Mean (95% CI)^e^	*p* Value	Mean (95% CI)^e^	*p* Value
Lumbar spine	1.18 (1.06, 1.32)	0.003	1.21 (1.07, 1.38)	0.003
Femoral neck	1.22 (1.07, 1.41)	0.003	1.15 (0.99, 1.32)	0.05

^a^ Significant bone loss (yes/no) using a least significant change‐based threshold was defined as an annualized lumbar spine (LS) or femoral neck (FN) bone mineral density (BMD) decline rate that was faster than 1.23% per year in LS and 1.93% per year in the FN.

^b^ Significant bone loss (yes/no) using a distribution‐based threshold was defined as an annualized LS or FN BMD decline rate that was faster than 1.59% per year in the LS and 1.86% per year in the FN.

^c^ Risk ratio (95% confidence interval) for significant bone loss (at lumbar spine and femoral neck) per standard deviation increment in urinary N‐telopeptide (14.6 nM BCE/mM Cr) adjusted for age, menopause transition stage, race/ethnicity, body mass index, sample collection time, starting BMD, and study site.

^d^ Risk ratio (95% confidence interval) for significant bone loss (at lumbar spine and femoral neck) per standard deviation increment in urinary N‐telopeptide (13.5 nM BCE/mM Cr) adjusted for age, race/ethnicity, body mass index, sample collection time, starting BMD, and study site.

^e^ Risk ratio (95% confidence interval) for significant bone loss (at lumbar spine and femoral neck) per standard deviation increment in urinary N‐telopeptide (15.9 nM BCE/mM Cr) adjusted for age, race/ethnicity, body mass index, sample collection time, starting BMD, and study site.

Age, MT stage, race/ethnicity, and BMI also were independently associated with the probability of significant bone loss (data not shown). Early perimenopausal women were 49% and 50% more likely to be losing significant bone at the LS than premenopausal women (*p* < 0.01) using LSC‐ and distribution‐based thresholds for bone loss, after controlling for U‐NTX and the other covariates. The ability of U‐NTX (in combination with age, MT stage, race/ethnicity, and BMI) to identify women who began losing significant bone at the LS during the next 3 to 4 years (as measured by the area under the receiver operating characteristic [ROC] curve [AUC]) was 0.72 (for loss defined using the LSC‐based threshold) and 0.74 (for loss defined using the distribution‐based threshold).

In analyses stratified by MT stage, baseline U‐NTX measured in women who were premenopausal at SWAN baseline did not predict onset of significant bone loss over the next 3 to 4 years. In contrast, baseline U‐NTX measured in women who were early perimenopausal at SWAN baseline did strongly predict risk of significant LS (by LSC‐ [AUC 0.72] and distribution‐based thresholds [AUC 0.74]) and FN bone loss (LSC‐based threshold [AUC 0.72]) (Table 2).

### Descriptive statistics for repeated U‐NTX measures

The average number of U‐NTX observations in premenopause per participant was 2.1, with median of 1 and range of 1 to 7. The average number of observations in early perimenopause per participant was 3.8, with median of 4 and range of 1 to 8. A greater proportion of visits in early perimenopause were followed by significant LS and FN bone loss compared with visits in premenopause (Table 3).

**Table 3:** Descriptive Statistics^a^ for Repeated Measures Samples

	Premenopausal subjects *n* = 720 1386 observations	Early perimenopausal subjects *n* = 1033 877 observations
Age (years)	46.8 (2.7)	48.6 (2.8)
Race/ethnicity		
African American	237 (17.1)	522 (18.1)
White	724 (52.2)	1367 (47.5)
Chinese	199 (14.3)	432 (15.0)
Japanese	226 (16.3)	556 (19.3)
Body mass index (kg/m^2^)	26.6 (6.4)	27.0 (6.5)
N‐telopeptide, urine (nM BCE/mM Cr)	33.3 (16.0)	33.6 (18.2)
Annualized percent change in bone mineral density		
Lumbar spine (% per year)	−0.2 (1.2)	−0.9 (1.4)
Femoral neck (% per /year)	−0.2 (1.2)	−0.7 (1.4)
Losing from baseline to 3 to 4 years later (least significant change‐based threshold)^b^		
Lumbar spine	234 (17.0)	1058 (36.9)
Femoral neck	100 (7.3)	491 (17.2)
Losing bone from baseline to 3 to 4 years later (distribution‐based threshold)^c^		
Lumbar spine	171 (12.5)	839 (29.3)
Femoral neck	112 (8.1)	534 (18.7)

^a^ Count (percentage) for categorical variables; mean (standard deviation) for continuous variables.

^b^ Significant bone loss (yes/no) using a least significant change‐based threshold was defined as an annualized lumbar spine (LS) or femoral neck (FN) BMD decline rate that was faster than 1.23% per year in the LS and 1.93% per year in the FN.

^c^ Significant bone loss (yes/no) using a distribution‐based threshold was defined as an annualized LS or FN BMD decline rate that was faster than 1.59% per year in the LS and 1.86% per year in the FN.

### Repeated measures analysis stratified by MT stage

In mixed‐effects, modified Poisson regression, after adjusting for age, BMI, race/ethnicity, urine collection time, starting BMD, and study site, premenopausal U‐NTX was not associated with risk of onset of significant bone loss over the next 3 to 4 years (Table 4). In contrast, in early perimenopause, greater U‐NTX was associated with greater risk of significant LS bone loss. Each SD increment in early perimenopausal U‐NTX increased risk of bone loss at the LS by 7% (*p* < 0.02) regardless of which BMD decline rate threshold was used to define bone loss.

**Table 4:** Associations^a^ Between Urinary N‐Telopeptide and Significant Bone Loss Over Next 3 to 4 Years, Stratified by MT Stage; Repeated Measures Analyses

	Bone loss by least significant change‐based threshold^b^		Bone loss by distribution‐based threshold^c^	
Premenopausal observations (*n* = 720 subjects, 1386 observations)				
	Mean (95% CI)^d^	*p* Value	Mean (95% CI)^d^	*p* Value
Lumbar spine bone loss	1.03 (0.95, 1.12)	0.4	1.01 (0.92, 1.12)	0.7
Femoral neck bone loss	1.07 (0.93, 1.22)	0.3	1.06 (0.92, 1.20)	0.4
Early perimenopausal observations (*n* = 1033 subjects, 2877 observations)				
	Mean (95% CI)^e^	*p* Value	Mean (95% CI)^e^	*p* Value
Lumbar spine bone loss	1.07 (1.01, 1.13)	0.002	1.07 (1.02, 1.13)	0.02
Femoral neck bone loss	1.07 (0.99, 1.16)	0.07	1.06 (0.99, 1.15)	0.1

^a^ Results of mixed‐effects, modified Poisson regression with robust estimation of standard error, using repeated measures of U‐NTX and bone loss.

^b^ Significant bone loss (yes/no) using a least significant change‐based threshold was defined as an annualized lumbar spine (LS) or femoral neck (FN) bone mineral density (BMD) decline rate that was faster than 1.23% per year in the LS and 1.93% per year in the FN.

^c^ Significant bone loss (yes/no) using a distribution‐based threshold was defined as an annualized LS or FN BMD decline rate that was faster than 1.59% per year in the LS and 1.86% per year in the FN.

^d^ Risk ratio for lumbar spine or femoral neck bone loss (yes/no) per standard deviation increment in premenopausal urinary N‐telopeptide (16.0 nM BCE/mM Cr) adjusted for age, ethnicity, body mass index, sample collection time, starting BMD, and study site.

^e^ Risk ratio for lumbar spine or femoral neck bone loss (yes/no) per standard deviation increment in early perimenopausal urinary N‐telopeptide (15.2 nM BCE/mM Cr) adjusted for age, ethnicity, body mass index, sample collection time, starting BMD, and study site.

The ability of early perimenopausal U‐NTX (in combination with age, race/ethnicity, and BMI) to identify those who would begin losing bone at the LS (as measured by AUC) was 0.72 (for both distribution‐ and LSC‐based thresholds). Given emerging data that the optimal way to utilize bone turnover markers is to incorporate them into prediction models that also include relevant clinical covariates,^9^ we created a “sample” tool that calculates the predicted probability of significant LS bone loss over the next 3 to 4 years (using the LSC‐based threshold) from user‐specified entries for race/ethnicity, age, BMI, and U‐NTX (Supplemental Data). Thus, for a white female who is 47 years of age, has a BMI of 22, and a U‐NTX of 32 BCE/mM Cr, the predicted probability of experiencing a significant bone loss at the LS over the next 3 to 4 years is 32%. If the same woman has a U‐NTX of 65 BCE/mM Cr, the predicted probability would be 85%.

### Sensitivity analyses

For each of the analyses described above, we conducted corresponding sensitivity analyses in which significant bone loss was defined as a BMD decline rate that was faster than the LSC defined as absolute change in BMD per year (0.016 g/cm^2^ per year for LS and 0.017 g/cm^2^ per year for FN). The proportions of women who were considered to be losing bone at the LS and FN using these thresholds were similar to those when the LSC was expressed as percent change in BMD per year. The association of U‐NTX with significant bone loss was also strongest at the LS during early perimenopause (data not shown).

## Discussion

The objective of this study was to answer two questions: 1) Can U‐NTX measured when a woman is in her mid‐40s to early 50s help determine if she is about to experience significant MT‐related bone loss; and 2) Is the ability of U‐NTX to predict the onset of MT‐related bone loss different when measured in pre‐ versus early perimenopause. We found that 42‐ to 52‐year‐old women with higher levels of U‐NTX were more likely to experience significant bone loss at the LS over the next 3 to 4 years than same‐aged women with lower levels of U‐NTX, but this association was driven entirely by women in early perimenopause. Premenopausal U‐NTX was not a predictor of significant bone loss over the next 3 to 4 years, whereas early perimenopausal U‐NTX did strongly predict bone loss. This was confirmed in repeated measures analyses using all premenopausal and early perimenopausal U‐NTX measurements from baseline and follow‐up visits. These repeated measures analyses allow for clinical situations wherein a woman may be evaluated anywhere from a few months to several years into the same MT stage. Combining early perimenopausal U‐NTX with relevant clinical covariates (age, race/ethnicity, and BMI) provided good discrimination between women who were more versus less likely to begin losing significant bone at the LS over the next 3 to 4 years (AUC >0.7).

Prior studies have examined the association between BTMs and *rates* of bone loss (as a continuous outcome). The totality of the literature suggests that greater levels of bone resorption markers are associated with faster bone loss.^9^, ^15^, ^16^, ^17^, ^18^, ^19^, ^20^, ^21^, ^22^, ^23^ The majority of these studies, however, were in women who were postmenopausal.^15^, ^17^, ^19^, ^21^ and had already experienced substantial BMD decline. In SWAN, we previously demonstrated that higher U‐NTX is associated with higher rates of BMD decline (continuous outcome) during the MT and early postmenopause.^9^ Here, we tested the ability of U‐NTX, measured early in the MT (ie, *in advance* of significant bone loss) to predict whether a woman would experience significant MT‐related bone loss (as a categorical outcome) over the next several years in a large, multi‐ethnic cohort. Recognizing that a woman is about to, or has just begun to, experience significant bone loss is one important consideration when deciding whether to initiate interventions to prevent rapid bone loss across the MT.

Our finding that early perimenopausal U‐NTX predicts the onset of MT‐related bone loss at the LS independent of age suggests that U‐NTX provides information about whether significant LS bone loss will occur in the next few years, above and beyond chronological age and clinical bleeding patterns. MT‐related bone loss begins approximately 1 year before the FMP.^2^ However, clinical bleeding patterns are not useful for predicting how many years a woman is from her FMP.^2^, ^24^ For example, women can experience anywhere from several months to more than 5 years of less predictable menstrual bleeding before having her FMP.^24^ In the absence of knowledge about how far a woman is from her FMP, U‐NTX can be valuable as a physiologic marker of imminent bone loss. This is suggested by our finding that although mean U‐NTX was similar during pre‐ and early perimenopause, there were more women in early perimenopause who had higher U‐NTX levels and were losing significant BMD.

The combination of early perimenopausal U‐NTX, age, race/ethnicity, and BMI (all independent predictors of bone loss) also provided good discrimination ability for onset of LS bone loss, as suggested by AUC values that exceed 0.7.^25^, ^26^ Consistent with the emerging consensus that the optimal way to utilize BTMs is to incorporate them into prediction models that also include relevant clinical covariates,^9^ we created an online tool that calculates the model‐predicted probability of significant LS bone loss over the next 3 to 4 years from user‐specified entries for race/ethnicity, age, BMI, and U‐NTX (Supplemental Data). We did not include BMD in this tool because clinicians may be reluctant to obtain a DXA scan in pre‐ and early perimenopausal women. Before it can be adopted for clinical use, the prediction tool needs to be externally validated and should ideally be recreated and validated using serum C‐terminal telopeptide of type I collagen in serum (S‐CTX), which is now recommended as the referent bone resorption marker.^27^

We also report here that significant bone loss was more likely to occur in the LS than in the FN, consistent with previous observations that the majority of bone remodeling early in the MT take place at trabecular surfaces, of which there are more in the LS than the FN.^28^, ^29^, ^30^, ^31^ This is also consistent with our finding that BMD declined faster at the LS than at the FN, and with previous findings that total BMD decline across the MT is greater at the LS than at the FN.^2^ These differences by site may explain why we found U‐NTX to be more strongly associated with bone loss at the LS than at the FN. We theorize that the association between U‐NTX and onset of FN bone loss was weaker because decline in FN BMD early in the MT was not substantially faster than the precision of DXA‐based FN BMD measurements.^32^ Along these lines, it is possible that U‐NTX would have more strongly predicted significant bone loss at the total hip because of lower variability. However, we did not have total hip BMD data in SWAN.

Our study has several limitations that should be noted. First, S‐CTX is now recommended as the referent bone resorption marker to use in clinical studies;^27^ however, SWAN, which started 20 years ago, measured U‐NTX.^33^ Additionally, urinary measures of bone turnover markers are criticized for being more variable than serum measures because the need to adjust for urinary creatinine introduces an additional source of variability.^34^ Given that we were able to detect a robust association between U‐NTX and onset of significant LS bone loss, we hypothesize that a bone resorption marker with less pre‐analytical variability would be better. A second limitation is that specimen storage temperature at local sites during the 1‐month period before shipment to Central Lab was not recorded in SWAN; we were therefore unable to control for this covariate in our analyses. Third, although every attempt was made to collect urine samples during the follicular phase of the menstrual cycle, this was not always feasible, especially in perimenopausal subjects with less predictable menstrual bleeding (up to 90 days between cycles). This may have increased the pre‐analytical variability of U‐NTX measurements.^34^ Finally, although combining U‐NTX with a bone formation marker improves prediction of rate of BMD decline,^35^ we could not test this approach in this study because we did not have enough contemporaneous measures of U‐NTX and a bone formation marker in SWAN.

To conclude, this study confirms that U‐NTX measured during early perimenopause is strongly associated with the likelihood of bone loss onset at the LS over the next several years, independent of age. In addition, its ability to identify women who are about to, or have just begun to, experience significant LS bone loss is robust when combined with relevant covariates. Future studies will determine the best bone turnover marker or best combination of markers needed to determine when women are about to begin rapid loss in bone mass and deterioration in microarchitecture before substantial skeletal deterioration. This, in turn, will allow us to test the long‐term efficacy of early interventions for preventing bone loss and microarchitectural damage.^9^

## Disclosures

JCL has received prior research funding from Amgen and current research funding from Sanofi Inc., unrelated to this study. AS, GAG, JAC, CKG, and ASK have nothing to disclose.

## Supporting information

Supporting Information S1.
